# Multi-potent rhizobacteria enhance banana growth and reduce chemical fertilizer input

**DOI:** 10.3389/fmicb.2025.1659278

**Published:** 2025-09-18

**Authors:** Jeberlin Prabina Bright, Kavitha Chinnasamy, Hemant S. Maheshwari, Kahkashan Perveen, Faheema Khan, Jayanthi Barasarathi, Balachander Dananjeyan, Nazih Y. Rebouh

**Affiliations:** ^1^Department of Soil Science and Agricultural Chemistry, VOC Agricultural College and Research Institute, Tamil Nadu Agricultural University, Thoothukudi, India; ^2^Turmeric Research Centre, Tamil Nadu Agricultural University, Erode, India; ^3^University of Groningen, Groningen, Netherlands; ^4^Department of Botany and Microbiology, College of Science, King Saud University, Riyadh, Saudi Arabia; ^5^Faculty of Health and Life Sciences (FHLS), Inti International University, Nilai, Malaysia; ^6^Department of Agricultural Microbiology, Tamil Nadu Agricultural University, Coimbatore, India; ^7^Department of Environmental Management, Institute of Environmental Engineering, RUDN University, Moscow, Russia

**Keywords:** banana, potassium solubilizing bacteria (KSB), potassium, savings of chemical fertilizers, solid and liquid formulations

## Abstract

**Introduction:**

The present investigation isolated efficient potassium-solubilizing bacteria (KSB) from the banana rhizosphere and, along with nitrogen- and phosphorus-transforming strains from TNAU, evaluated their impact on banana growth, yield, and fertilizer reduction.

**Methods:**

Solid and liquid-based formulations using the nitrogen-fixing bacterium *Azospirillum brasilense* Sp7 (*A. b* Sp7), phosphorus-solubilizing bacterium *Bacillus megaterium* Pb1 (*B. m* Pb1), and the newly isolated potassium-solubilizing bacteria *Agrobacterium pusense* (*A. p*. KRBKKM1) and *Bacillus paralicheniformis* (*B. p* KRBKKM2) were prepared and used for inoculation in the field experiment. HPLC profiling of KSBs showed that *A. p*. KRBKKM1 produced propionic acid, and *B. p* KRBKKM2 produced butyric and propionic acids.

**Results:**

Among the two formulations tested, the liquid formulation had a significantly greater influence on the yield and yield-attributing traits than the solid-based ones. Treatments consisted of 75% NPK chemical fertilizer + 2 mL *A. b* Sp7 + 2 mL *B. m* Pb1, along with 1 mL *A. p*. KRBKKMI and 1 mL *B. p* KRBKKM2 (T10-30.65 t ha^−1^), and 75% NPK + 2 mL *A. b* Sp7 + 2 mL *B. m* Pb1, along with 2 mL *A. p*. KRBKKMI and 2 mL *B. p* KRBKKM2 (T11-30.82 t ha^−1^) significant impacted the banana yield parameters.

**Discussion:**

Principal component analysis revealed that treatments T10 and T11 positively correlated with yield-related parameters. Pearson correlation analysis revealed that crop yield was positively correlated with the bunch weight (*r* = 1.00***), fruit weight (*r* = 0.84**), and number of fingers per bunch (*r* = 0.76**), in both the solid and liquid formulations. In conclusion, the present investigation demonstrated a 25% reduction in chemical inputs when using NPK biofertilizers and contribute to increased agricultural productivity.

## Introduction

Banana (*Musa* spp.) belongs to the Musaceae family and requires comparatively higher macro and micronutrients than other crops. Recommended doses of macronutrients through chemical fertilizers for bananas are 110:35:330 g NPK per plant, applied in three split doses at 3, 5, and 7 months to achieve higher yields^1^ in Indian soils, which vary with soil type and nutrient content. The Food and Agriculture Organization of the United Nations (2022) report identified banana toxic residue levels as a key concern limiting banana exports.^2^ Utilizing potent microbes as biofertilizers would be the awaited and holistic solution to meet crop nutrient demand by replacing partial doses of chemical fertilizers (Pradhan et al., 2025). Among the nutrients, bananas highly demand potassium for their growth, followed by nitrogen (Villasenor-Ortiz, 2022).

Plant-bound potassium in plant tissue metabolizes young, developing, and reproductive organs. It acts as a macronutrient, a cross-communicating molecule, an osmoticum, and a plant signaling molecule. It activates more than 60 plant enzymes and tolerates abiotic and biotic stresses in crops. Further, it interacts with plant growth hormones, reactive oxygen species, and other plant nutrients (Pandey and Mahiwal, 2020).

Though potassium is the seventh most abundant element in the Earth’s crust, approximately 90–98% is present in unavailable form as this cation is sandwiched between the clay mineral layers or gets complexed with other ions and becomes insoluble (Zorb et al., 2014; Volf et al., 2018; Olaniyan et al., 2022). Therefore, despite its abundance in the soil, the availability of potassium from the soil to the plants is limited. Thus, potassium availability from minerals depends on the soil-inhabiting bacteria that transform potassium (Kumar et al., 2020).

Potassium-solubilizing bacteria (KSB) solubilize and transform insoluble potassium by acidification via releasing protons and through various organic acids such as malic, succinic, citric, 2-ketogluconic, fumaric, oxalic, tartaric, glycolic, and lactic acids, and inorganic acids (Ali et al., 2021; Soumare et al., 2023; Baba et al., 2021; Meena et al., 2015). These acids solubilize insoluble potassium from igneous and sedimentary origin minerals into a plant-available form. Further, KSB enhanced plant health by utilizing direct and indirect plant growth-promoting actions against various abiotic and biotic stresses (Imran et al., 2020; Kaur et al., 2021; Khan et al., 2021; Mahala et al., 2020; Olaniyan et al., 2022; Bright et al., 2022). Key genera involved in potassium solubilization are *Bacillus*, *Enterobacter*, *Pantoea*, *Klebsiella*, *Stenotrophomonas*, *Microbacterium*, *Azotobacter*, *Paenibacillus*, *Acidithiobacillus*, *Pseudomonas*, *Burkholderia*, *Rhizobium pusense*, *Flavobacterium*, and *Agrobacterium* (Meena et al., 2015; Raghavendra et al., 2016; Teotia et al., 2016; Kour et al., 2020).

The level of chemical fertilizers and biofertilizers’ formulation influences the biocompatibility and the effect of biofertilizers on the soil (Sagar et al., 2022; Jabborova et al., 2025a, 2025b; Khan et al., 2025). Therefore, the present study aimed to link these concerns of reducing inorganic fertilizers with NPK bio-fertilizers and to validate the optimum NPK bio-fertilizer dosage for the banana crop and the best-suited formulation. To develop a microbial formulation for bananas, two new potassium-solubilizing bacteria have been isolated from the banana rhizosphere. Their effects on banana yield parameters and yield have been investigated through a field study, where they were formulated as NPK consortia along with nitrogen-fixing *Azospirillum brasilense* Sp7 and phosphorus-solubilizing bacteria *Bacillus megaterium* Pb1.

## Materials and methods

### Isolation of potassium-solubilizing bacteria from banana rhizosphere soil

A hundred different soil samples were collected from the rhizosphere of banana crops grown in various locations of Thoothukudi district [8.764166°N, 78.134834°E**]** of Tamil Nadu, India. Potassium-solubilizing bacteria (KSB) were isolated from the collected soil samples using the serial dilution and plating technique with the selective Aleksandrov agar medium (Aleksandrov et al., 1967). Bromothymol blue was added as a pH indicator in the medium. The plates were incubated at 30 ± 2 °C for 5 days. Bacterial colonies that exhibited the highest clearing zone by solubilizing the insoluble potassium and the medium’s color change from greenish blue to yellow were selected (Parmar and Sindhu, 2018). Two morphologically distinct isolates, KRBKKM1 and KRBKKM2, from Srivaikundam and Murappanadu in Thoothukudi district, Tamil Nadu, India, respectively, were selected and purified using the quadrant streaking method for further studies.

### Characterization and identification of the potassium-solubilizing bacterial isolates

Colony characteristics were studied, including size, elevation, color, margin, pigmentation, and Gram reaction. Furthermore, Gram reaction, carbohydrate utilization, and other biochemical characteristics of KSB were examined, as mentioned by Cappuccino and Sherman (2014).

The potassium solubilization efficiency (KE) of the bacterial isolates was estimated as a physical measure of their ability to release potassium by spotting the bacterial isolates onto Aleksandrov agar containing potassium alumino-silicate in three replicates. The diameter of the zone of solubilization was measured on the third day after inoculation, and the KE was calculated (Ramesh et al., 2014). A positive control from the TNAU culture collection (*Paenibacillus mucilaginosus* KRB9) was maintained to compare the efficiency in solubilizing potassium under *in vitro* conditions.

$$\mathrm{KE}=\frac{\text{Diameter of solubilization halo zone}+\text{Diameter of colony}}{\text{Diameter of the colony}}$$

The bacterial isolates KRBKKM1 and KRBKKM2 were further tested for their ability to release potassium from insoluble mica under in vitro conditions, as indicated by the amount of potassium released and the change in pH of the medium. One milliliter of overnight-grown cultures of each bacterial isolate was inoculated into 25 mL Aleksandrov broth supplemented with 1% potassium alumino-silicate (mica) and incubated at 30 °C for 20 days. For each isolate, three replicates were maintained. The suspension was centrifuged at 15,000 rpm for 20 min, and the supernatant was filtered through a membrane (Francis et al., 1988) and quantified using a flame photometer. The amount of potassium released was estimated on 3, 7, 12, 16, and 20 days of inoculation. The change in pH of the medium was recorded using a pH meter (Sigma-Aldrich—Acorn) on days 7, 12, 16, and 20 of the inoculation period.

### Detection of organic acids produced by the KSBs through HPLC

The KSB isolates were inoculated separately in Aleksandrov broth supplemented with 1% mica in triplicate. The culture flasks were incubated in an orbital shaker (REMI-CIS 24 plus TFT) at 37 °C with intermittent short span shaking at 100 rpm for 15 min a day for 16 days. One milliliter of the suspension from the flask was centrifuged at 10,000 rpm for 15 min, and the supernatant was filtered using a 0.22 μm nylon membrane filter (Sigma-Aldrich). The resultant cell-free supernatant was used for HPLC analysis. Twenty microlitres of the supernatant were injected onto the column of the HPLC (LC-10AT Shimadzu). An ion-exclusion Aminex column (HPX-87H, 300 mm × 7.8 mm, Bio-Rad) was used for chromatographic separation. A 0.008 M H_2_SO_4_ solution, prepared with HPLC-grade distilled water, was used as the mobile phase, with a constant flow rate of 0.6 mL min^−1^ and an operating temperature of 30 °C. A UV–VIS detector was used to record the retention times of the compounds at 210 nm, and an organic acids analysis standard kit (Bio-Rad) was used as a known standard for comparison (Yadav et al., 2013). The organic acids were identified based on the comparison of the retention time of the samples and the standard.

### Characterization of plant growth-promoting traits of the KSB isolates

#### Qualitative assessment of exopolysaccharide production

The two bacterial isolates, KRBKKM1 and KRBKKM2, were tested for their ability to produce exopolysaccharides by spotting over glucose minimal agar medium in three replicates, as mentioned in Vijayabaskar et al. (2011). The amount of polysaccharide produced was observed after 48 h and scored as no polysaccharide (−), weak (+), moderate (++), and high polysaccharide production (+++).

#### Siderophore production

The siderophore-producing ability of the potassium solubilizing isolates was tested in three replications with a modified chrome azurol sulphonate (CAS) agar assay (Milagres et al., 1999; Patel et al., 2018). CAS blue agar and Aleksandrov medium supplemented with potassium as 1% potassium chloride were solidified onto Petri plates. One-half of the CAS blue agar was replaced with Aleksandrov medium. The potassium-solubilizing bacterial isolate was streaked onto Aleksandrov medium along the border between the two media and incubated in the dark at 30 ± 2 °C for 10 days. An uninoculated CAS agar plate was maintained as a control by placing it in the dark under the same conditions. Siderophore production was confirmed based on the change in the color of the CAS blue agar medium from blue to yellow along the borderline (Schwyn and Neilands, 1987).

#### Qualitative estimation of zinc solubilization

Both the KSB isolates were tested for zinc solubilization, and the solubilization index was recorded. The zinc solubilization ability of the KSB isolates was tested in three replications as described in Sharma et al. (2012) using a Tris-minimal medium amended with 0.5% D-glucose and 0.1% zinc oxide. The plates were spotted with KRMKKM1 and KRBKKM2 separately and incubated at 30 ± 2 °C for 7 days. The clearing zone around the colonies and the diameter of the colonies were measured to calculate the zinc solubilization index (Sethi et al., 2025). Further methyl red was added to the plates to confirm acid production by the isolates.

$$\mathrm{SI}=\frac{\left(\text{Diameter of solubilization halo zone}+\text{diameter of the colony}\right)}{\text{Diameter of the colony}}$$

#### Identification of the potassium-solubilizing bacterial isolates based on 16S rDNA and phylogenetic analysis

The genomic DNA was isolated by the CTAB method (Atashpaz et al., 2010), and further, the genomic DNA was amplified using the universal primers 27 F 5′AGAGTTTGATCCTGGCTCAG 3′ and reverse 1492 R 5′ GGTTACCTTGTTACGACTT 3′ primers. The PCR program was set up as follows: initial incubation at 94 °C for 5 min, followed by 35 cycles (94 °C for 1 min, 52.5 °C for 1 min, and 72 °C for 2 min), and final extension at 72 °C for 10 min using a thermal cycler. A single discrete PCR amplicon band was obtained when resolved on an agarose gel. Consensus sequences of the 16S rDNA were generated from the forward and reverse primers using the aligner software. The BLAST search compared the resulting 16S rDNA gene sequence with the NCBI data. The generated sequences of KRBKKM1 and KRBKKM2 were analyzed using the NCBI BLAST tool, and the isolates were identified based on homology. Sequences showing high similarity were selected for phylogenetic analyses. Sequences were aligned using MUSCLE, and phylogenetic analysis was performed with the MEGA10 software package.^3^ The evolutionary history was inferred through the maximum likelihood method with 1,000 bootstrap replicates (Tamura et al., 2004).

#### Experimental site and experimental details

The field experiment was conducted under garden land conditions at VOC Agricultural College & Research Institute, Killikulam (8.71021°N, 77.85545°E), Thoothukudi district, with Banana cv. Rasthali (AAB) in a randomized block design. Banana sword suckers, 2 months old and weighing about 1–1.5 kg, were used as the planting material. The roots were trimmed, and the pseudostem was cut, leaving 20 cm from the corm. Then, the suckers were disinfected by dipping in a slurry solution containing 4 parts clay and 5 parts water, and sprinkling 40 g of carbofuran per corm to prevent nematode problems. The spacing adopted was 2.1 × 2.1 m, giving a banana plant density of 2,267 plants per hectare. This experiment was carried out in a total area of 0.5 hectares and was divided into two halves, with 0.25 hectares for each formulation.

Each half was divided into three equal blocks to accommodate three replications. Each block was divided into experimental units. Each experimental unit measured 8.4 m × 6.3 m and accommodated 20 plants. The treatment combinations were randomized among the blocks and within the blocks. Ten plants were tagged in each plot, observations were recorded for the tagged plants, and the mean was calculated. Each treatment was replicated thrice. The experiment was conducted in 2023, and the weather was tropical, with wet and dry conditions. The total crop period was 12 months. The average maximum temperatures ranged from 26.2 °C to 39.1 °C, and the minimum temperatures ranged from 21.9 °C to 22.2 °C. The precipitation during the crop period ranged from 0.0 to 10.9 mm. All recommended agronomic and management practices were followed per the standard recommendations.^4^

The soil of the experimental site was lateritic with a pH of 6.8, an EC of 0.12 dSm^−1^, an organic carbon content of 0.23%, a nitrogen of 199 kg ha^−1^, a phosphorus of 22 kg ha^−1^, and a potassium of 236 kg ha^−1^_._ The experimental field had a total bacterial count of 8 × 10^6^ CFU g^−1^ dry soil, a fungal count of 4 × 10^5^ CFU g^−1^ dry soil, and a total actinobacteria count of 5 × 10^5^ CFU g^−1^ dry soil before planting.

#### Preparation of solid and liquid biofertilizer formulations

The nitrogen-fixer *Azospirillum brasilense* Sp7, phosphorus-solubilizing bacteria *Bacillus megaterium* Pb1, and the newly isolated potassium solubilizers *Agrobacterium pusense* KRBKKM1 and *Bacillus paralicheniformis* KRBKKM2 were prepared as bio-fertilizers in solid and liquid formulations using a standard protocol^5^ for use in the field experiment. Lignite was used as the carrier material to prepare a solid formulation. For culturing of *Azospirillum brasilense* Sp7, Dobereiner’s malic acid broth (composition per litre-Malic acid–5 g; K_2_HPO_4_–0.5 g; MgSO_4_7H_2_O–0.2 g; NaCl–0.1 g; CaCl_2_–0.02 g; Fe EDTA 1.64% W/V aqueous-4 mL; Trace element solution-2 mL; Vitamin solution-1 mL; adjusted to pH-6.8 with 1 N KOH) supplemented with ammonium chloride 5 g L^−1^ broth was used as culturing medium. For culturing *Bacillus megaterium* Pb1, *Agrobacterium pusense* KRBKKM1, and *Bacillus paralicheniformis* KRBKKM2, nutrient broth (composition g L^−1^—peptone-5 g; beef extract-3 g; sodium chloride-5 g; adjusted to pH-7.0 with 1 N NaOH) was used. A loopful of mother culture was inoculated into the flasks containing the concerned culture medium. The flasks were kept under shaking conditions at 30 ± 2 °C till a cell population of 10^10^ to 10^11^CFU mL^−1^ was reached, and the incubation period was 5 to 7 days for *A. b* Sp7; 2 to 3 days for *B. m* Pb1, *A. p* KRBKKM 1, and *B. l* KRBKKM 2. The culture thus obtained in the flask is called the starter culture. The starter was inoculated at 5% concentrations, in large-sized flasks of 3 liters and grown until the required cell count level was reached (10^10^ to 10^11^ CFU mL^−1^). This is called the seed inoculum. Further mass culturing was done in a laboratory fermenter (Lark Innovative) with a capacity of 50 litres under controlled conditions with an intermittent air supply of 3–5 L per hour per litre of the medium. The broth was harvested with a population load of 10^−9^ cells mL^−1^ after incubation through the sampling port and mixed in a mechanical mixer with lignite at 400 mL kg^−1^. The mixed inoculum was cured for 2 days at 30 ± 2 °C and was used for inoculation in the experimental field. For liquid formulation after harvesting from the fermenter, glycerol was added at 5% concentration during bottling, which would prevent desiccation of bacterial cells and help uniform adherence of the inoculum to the substrate. After preparation, the population of bacterial cells in the solid (lignite) based formulation was 10^10^ colony-forming units CFU g^−1^ dry weight. The liquid formulation contained an inoculum load of 10^11^ CFU mL^−1^. The solid-based formulation’s shelf life is 6 months, and that of the liquid is 12 months. The bio-fertilizers were applied to the pit during planting and the fifth and seventh months. The experiment consisted of 22 treatments in 3 replications, conducted in a randomized block design (RBD), and the experiments were performed separately for solid (Table 1) and liquid biofertilizer formulations (Table 2).

**Table 1:** Treatment details of the present investigation using a solid (lignite) formulation.

Treatment	Details
T1	Untreated control-100% NPK (110:35:330 g NPK/plant)-(Negative control)
T2	100% NPK + 5 g *A. b* Sp7 + 5 g *B. m* Pb1 (Positive control I)
T3	100% NPK + 5 g *A. b* Sp7 + 5 g *B. m* Pb1 + 5 g *A. p* KRB KKM 1
T4	100% NPK + 5 g *A. b* Sp7 + 5 g *B. m* Pb1 + 5 g *B. l* KRB KKM 2
T5	100% NPK + 5 g *A. b* Sp7 + 5 g *B. m* Pb1 + 5 g KRB (2.5 g each of KRB KKM 1 & 2)
T6	100% NPK + 5 g *A. b* Sp7 + 5 g *B. m* Pb1 + 10 g KRB (5 g each of KRBKKM 1 & 2)
T7	75% NPK + 5 g *A. b* Sp7 + 5 g *B. m* Pb1 (Positive control II)
T8	75% NPK + 5 g *A. b* Sp7 + 5 g *B. m* Pb1 + 5 g *A. p* KRB KKM 1
T9	75% NPK + 5 g *A. b* Sp7 + 5 g *B. m* Pb1 + 5 g *B. l* KRB KKM 2
T10	75% NPK + 5 g *A. b* Sp7 + 5 g *B. m* Pb1 + 5 g KRB (2.5 g each of KRB KKM 1 &2)
T11	75% NPK + 5 g *A. b* Sp7 + 5 g *B. m* Pb1 + 10 g KRB (5 g each of KRB KKM 1 & 2)

**Table 2:** Treatment details of the present investigation using the liquid formulation.

Treatment	Details
T1	Untreated control-100% NPK (110:35:330 g NPK/plant)-(Negative control)
T2	100% NPK + 2 mL *A. b* Sp7 + 2 mL *B. m* Pb1 (Positive control)
T3	100% NPK + 2 mL *A. b* Sp7 + 2 mL *B. m* Pb1 + 2 mL KRB KKM 1
T4	100% NPK + 2 mL *A. b* Sp7 + 2 mL *B. m* Pb1 + 2 mL KRB KKM 2
T5	100% NPK + 2 mL *A. b* Sp7 + 2 mL *B. m* Pb1 + 2 mL KRB (1 mL each of KRBKKM 1 & 2)
T6	100% NPK + 2 mL *A. b*Sp7 + 2 mL *B. m* Pb1 per+4 mL KRB (2 mL each of KRBKKM 1 & 2)
T7	75% NPK + 2 mL *A. b* Sp7 + 2 mL *B. m* Pb1-(Positive control II)
T8	75% NPK + 2 mL *A. b* Sp7 + 2 mL *B. m* Pb1 + 2 mL KRB KKM 1
T9	75% NPK + 2 mL *A. b* Sp7 + 2 mL *B. m* Pb1 + 2 mL KRB KKM 2
T10	75% NPK + 2 mL *A. b* Sp7 + 2 mL *B. m* Pb1 + 2 mL KRB (1 mL each of KRB KKM 1 & 2)
T11	75% NPK + 2 mL *A. b* Sp7 + 2 mL *B. m* Pb1 + 4 mL KRB (2 mL each of KRBKKM 1 & 2)

The dosage is given per pit at the time of planting and on the 5th and 7th months after planting. *A. b* Sp7, *Azospirillum brasilense* Sp7; *B. m* Pb1, *Bacillus megaterium*; *A. p*. KRBKKM1, *Agrobacterium pusense*, and *B. p* KRBKKM2, *Bacillus paralicheniformis*.

The treatments were fixed to record the influence of two inorganic fertilizers (100 and 75%) levels, the newly isolated potassium solubilizing bacteria, and two biofertilizer formulations (solid and liquid). Full doses (100%) and half doses (75%) of NPK fertilizer without microbial inoculation were considered the negative control. Likewise, fertilizer doses of either 100% or 75% with nitrogen-fixing and phosphorus-solubilizing bacteria in solid (5 g) or liquid formulation (2 mL) were considered the positive control.

#### Recording banana yield parameters and yield

All parameters were recorded from the 10 tagged plants in each replication, and the mean value was calculated. The mean number of fingers per hand was calculated by counting the number of fingers in each hand and dividing by the total number of hands. The mean number of hands of the 10 tagged plants was divided by the number of bunches (Lamessa, 2021). The average weight of 10 uniformly ripe single fruits was measured in an electronic balance after separating the fingers from the middle hand. Additionally, for the same samples, the mean weight of pulp and peel was recorded separately and expressed in grams (Al-Harthi and Al-Yahyai, 2009). The bunch weight was measured using a platform balance, and the mean value was recorded. Yield per hectare was calculated by multiplying the mean weight of the bunch and the number of banana plants per hectare (2,267 nos.), and expressed in tons per hectare (Lamessa, 2021).

### Statistical analysis

One-way and two-way analysis of variance (ANOVA) were performed using IBM SPSS version 28 software at a significance level of 5% (*p* < 0.05). A multiple comparison analysis was done through Duncan’s multiple range test (DMRT). Graphs were prepared using GraphPad Prism software version 8.0.1. Further, principal component analysis (PCA) and Pearson correlation were carried out through R Studio version 4.4.1 using “Devtool,” “FactoMiner,” “Factoextra,” “ggplot2,” “metan,” and “corrplot.”

## Results

### Isolation and characterization of KSB isolates

**Figure 1:**
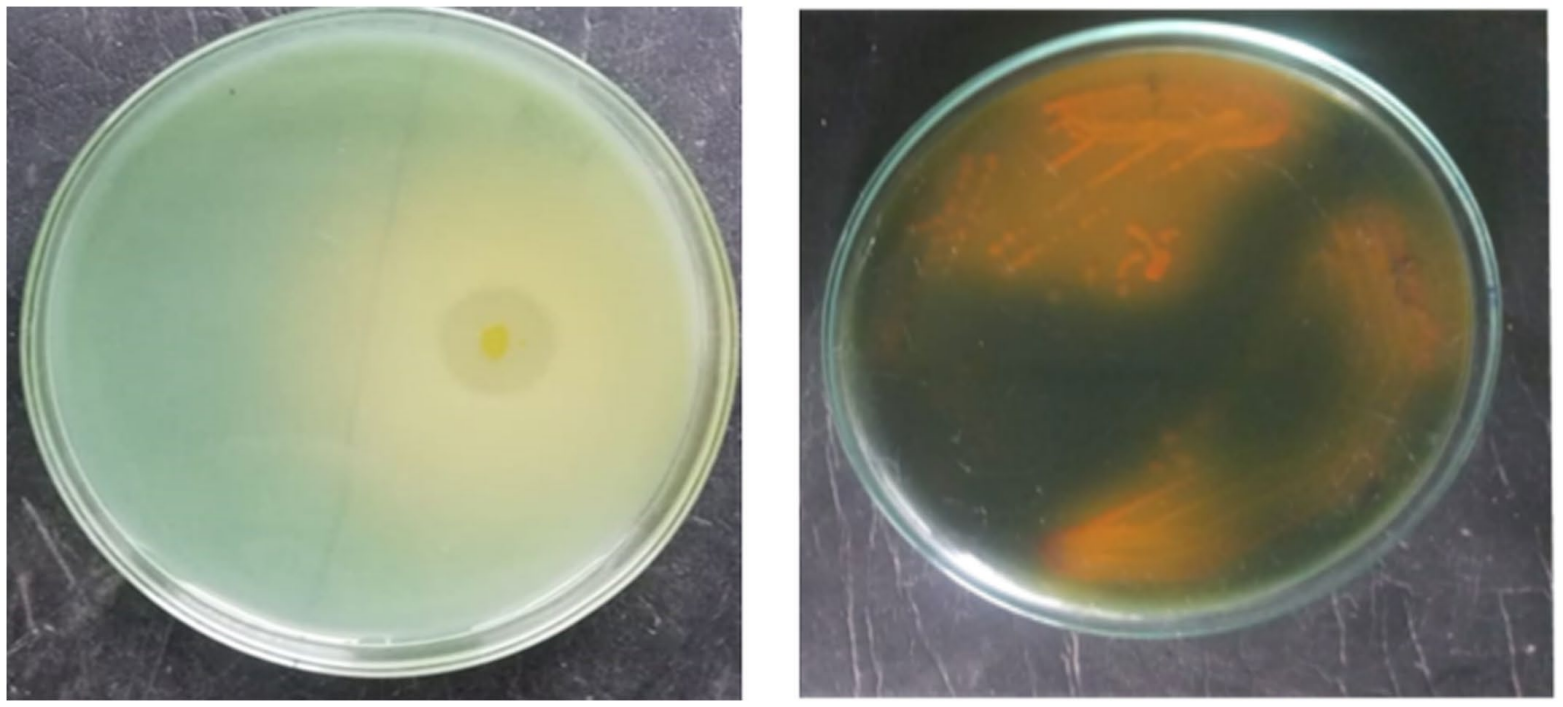
**(a)** Morphology of KRBKKM 1 showing KE; **(b)** showing colony morphology of KRBKKM 2.

A total of 24 morphologically different isolates were obtained from different rhizosphere soils of banana. Among them, initially, three morphologically different isolates (KRBKKM1, KRBKKM2, KRBKKM3) were selected that showed a higher solubilization zone on the 7th day after plating and a change in the color of the medium from greenish blue to yellow. The Potassium Solubilization Efficiency (KE) was 3.40, 4.33, and 3.12 for KRBKKM1, KRBKKM2, and KRBKKM3 isolates, respectively. Based on KE, KRBKKM1 and KRMKKM2 isolates were selected for further study (Figure 1a).

The colony of KRBKKM1 on Aleksandrov medium, after 3 days of inoculation, was of medium size, flat, cream-colored, and irregular margin with light orange pigmentation, which became more pronounced after prolonged incubation of 10 days. Similarly, KRBKKM2 was of medium size, raised, white, spreading, and irregular margin, with dark-orange pigmentation after prolonged incubation for 10 days following inoculation (Figure 1b).

Gram reaction showed KRBKKM1 as Gram-negative and KRBKKM2 as Gram-positive. Both isolates were rod-shaped and had a single-cell arrangement. The carbohydrate utilization test showed that KRBKKM1 and KRBKKM2 utilized glucose, fructose, sucrose, and maltose. However, both isolates failed to grow on cellulose-containing carbohydrates. Furthermore, both isolates were positive for the methyl red test, urease test, citrate utilization test, and catalase test. These KSB isolates were negative for the Voges–Proskauer and casein hydrolysis tests. Colony morphological characteristics and biochemical characteristics studied for phenotyping the potassium solubilizing bacterial isolates are presented in (Supplementary Table S1).

### Molecular characterization of the KSB isolates based on the 16S rDNA gene and phylogenetic analysis

**Figure 2:**
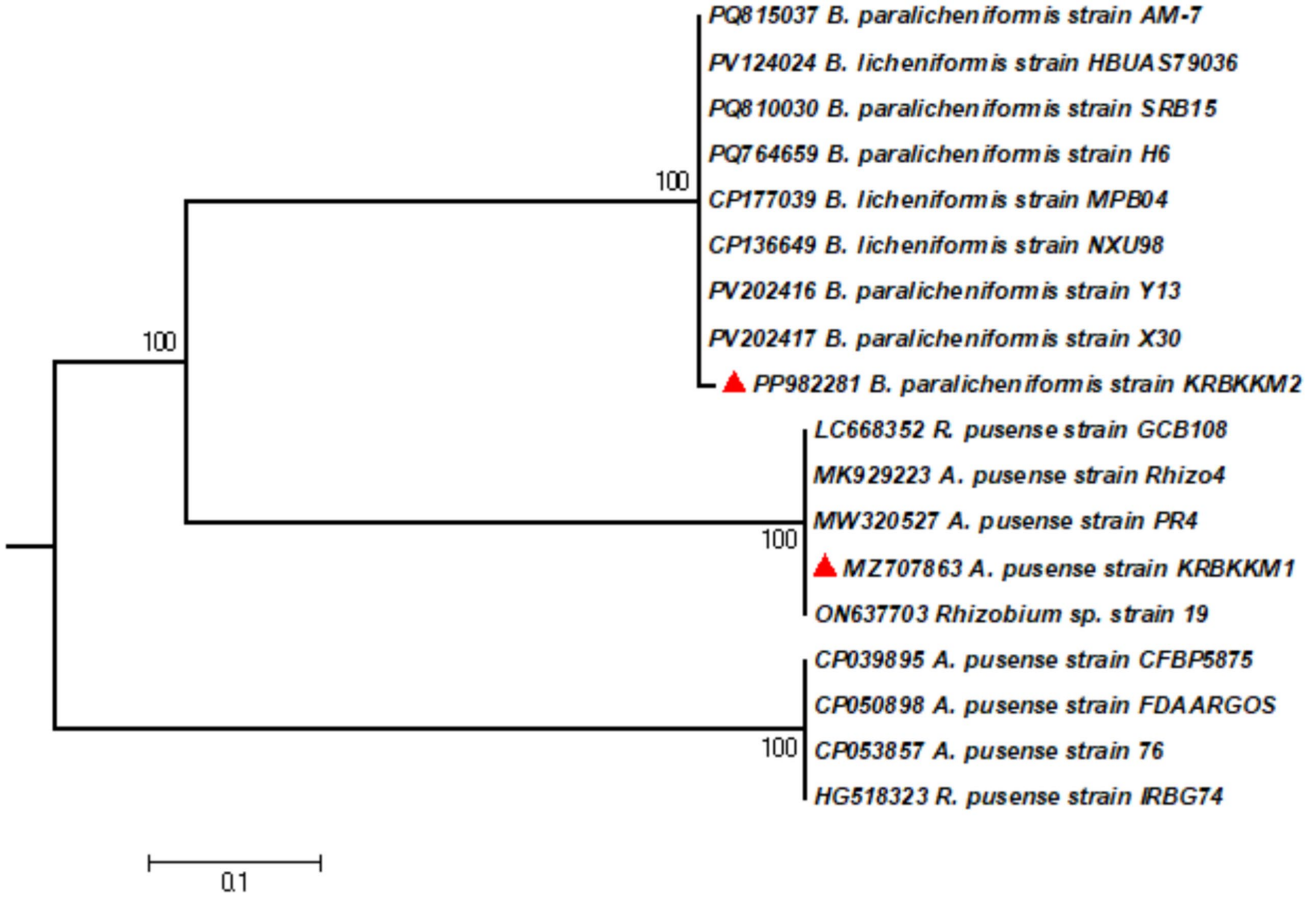
The phylogenetic tree of KRBKKM1 and KRBKKM2 was constructed with MEGA10 software using the maximum likelihood method with 1,000 bootstrap replicates.

NCBI-BLAST analysis was performed using the 16S rDNA sequence to ensure the isolates’ molecular-level identification. Based on the nucleotide homology followed by construction of a phylogenetic tree (Figure 2) using MEGA10 software, KRBKKM1 was identified as *Agrobacterium pusense* (Genbank accession No. MZ707863.1) and KRBKKM2 as *Bacillus paralicheniformis* (Genbank accession No. PP982281.1).

### Determination of potassium solubilization efficiency (KSE) and quantitative estimation of potassium released by the KSB isolates

The potassium solubilizing efficiency of the KRBKKM1 and KRBKKM2 isolates was comparable to that of the positive control, TNAU-KRB9 isolates (Table 3). Regarding the release of potassium from the insoluble potassium source, mica, a two-way interaction was observed, showing that the quantitative release of potassium by KRBKKM2 was significantly (*p* < 0.001) higher than that of KRBKKM1, and the least was observed with the positive control TNAU-KRB9 isolates (Table 3). Moreover, irrespective of the potassium solubilizing isolates, the rate of potassium released by the bacterial isolates increased as the days progressed until 16 days of inoculation and then decreased further (Table 4). Additionally, the interaction between potassium solubilizing isolates and the number of days of incubation was significant (*p* < 0.001). The pH of the inoculated medium was seven on day 0 of inoculation and subsequently decreased to 5.9. 4.2, 3.9, 2.5 and 2.1, respectively, on 7th, 12th, 16th, and 20th days of inoculation, respectively.

**Table 3:** Potassium solubilization efficiency (KE) and quantity of potassium released by the bacterial isolates at different intervals.

Bacterial isolates	KE	Potassium released (mgL^−1^)
KRB KKM1	3.40 ± 0.05a	73.08 ± 31.24b
KRB KKM2	4.43 ± 0.28a	80.10 ± 34a
Control-KRB TNAU-KRB	3.67 ± 0.57a	68.72 ± 26.72c

Value is the average of triplicates (*n* = 3) ± standard deviation. Different letters indicate significant differences at *p* < 0.05.

**Table 4:** Two-way interaction of inorganic potassium released by the potassium-solubilizing bacterial isolates recorded at different intervals.

Potassium-solubilizing bacterial isolates	Amount of inorganic potassium released (mgL^−1^) days of inoculation
3D	28.28e
7D	53.52d
12D	83.17c
16D	109.41a
20D	94.89b

### HPLC profiling of organic acids released by KSBs during the solubilization of mica

**Figure 3:**
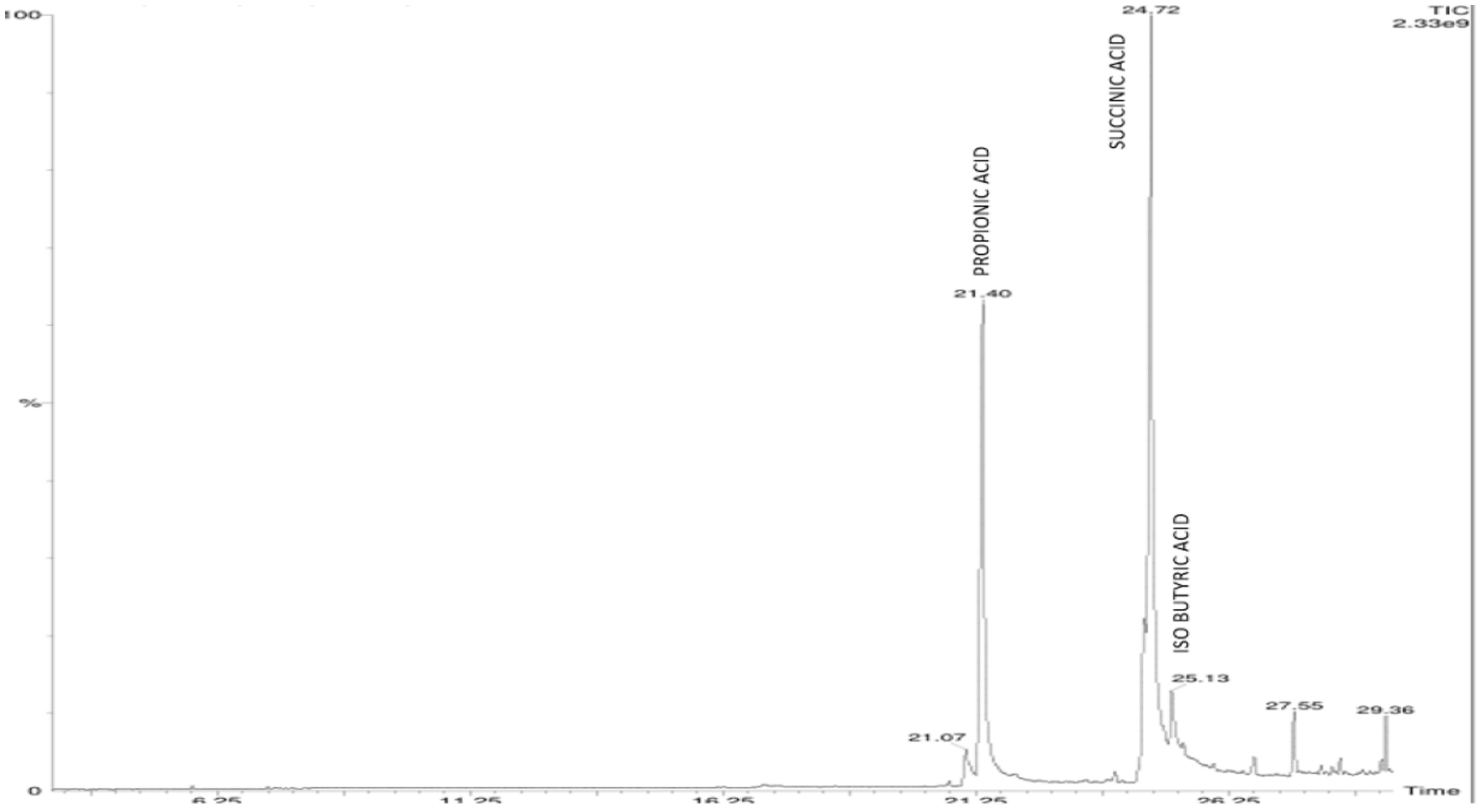
HPLC chromatogram of organic acids of *Agrobacterium pusense* KRBKKM1 in Aleksandrov broth supplemented with mica 16 days after incubation.

**Figure 4:**
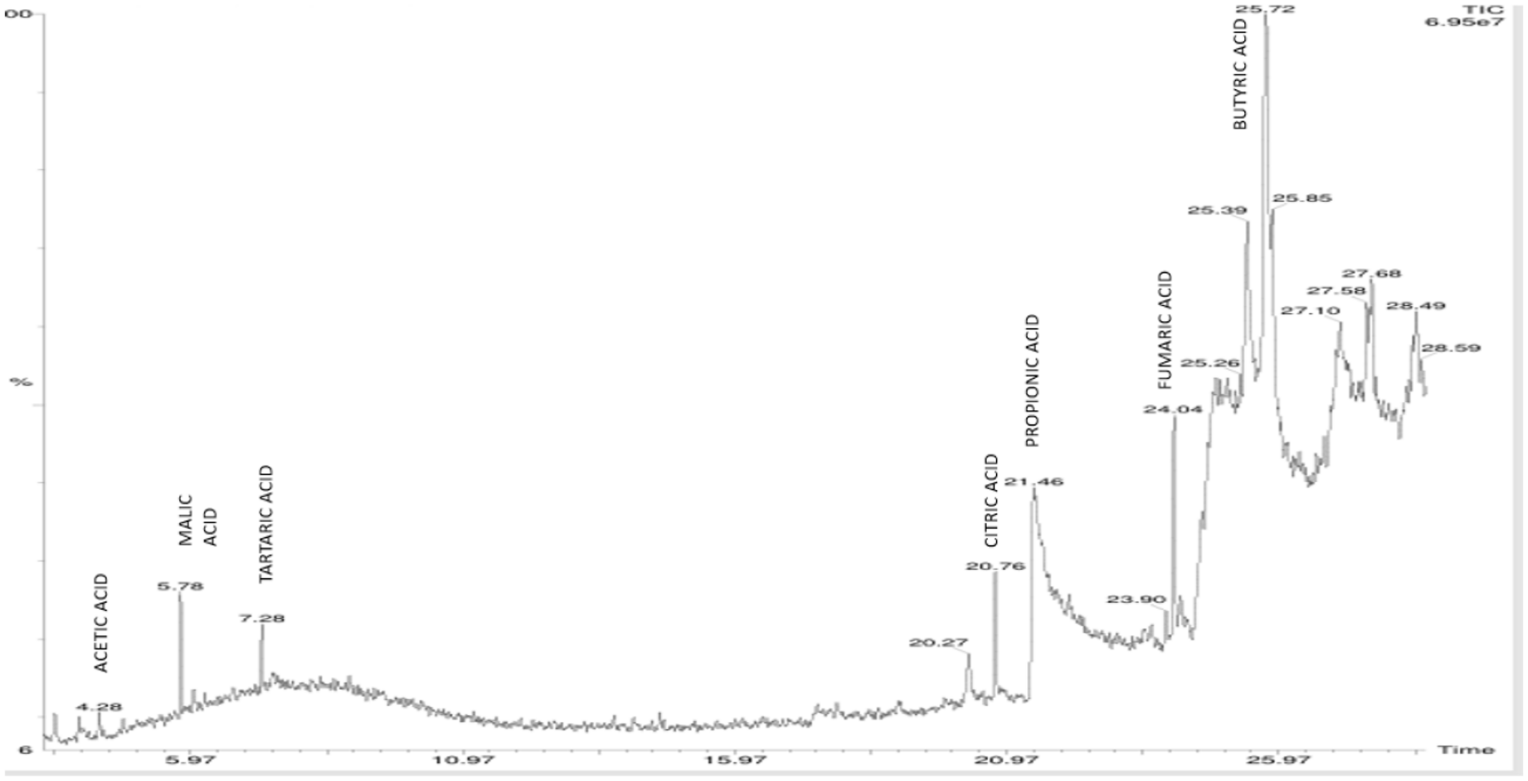
HPLC chromatogram of organic acids of *Bacillus licheniformis* KRBKKM2 in Aleksandrov broth supplemented with mica on 16 days after incubation.

On elution of the cell free supernatant of *Agrobacterium pusense* KRBKKM1 on 16^th^ day after inoculation in medium supplemented with mica as insoluble source of potassium, the organic acids namely propionic acid (RT 21.40), succinic acid (RT 24.72) and isobutyric acid (RT 25.13) were detected (Figure 3). Similarly, in *Bacillus licheniformis* KRBKKM2, acetic acid (RT 4.28), malic acid (RT 5.78), tartaric acid (RT 7.28), citric acid (RT 20.76), propionic acid (RT 21.46), fumaric acid (RT 24.04) and butyric acid (RT 25.72) were detected (Figure 4). The production range of organic acids was more in *Bacillus licheniformis* KRBKKM2 than in *Agrobacterium pusense* KRBKKM1.

### Plant growth-promoting traits of the potassium-solubilizing bacterial isolates

Both isolates, KRBKKM1 and KRBKKM2, exhibited multiple plant growth-promoting traits. Exopolysaccharide production was tested to confirm the isolates’ ability to colonize the rhizosphere region. The isolate KRBKKM1 excreted a moderate quantity of polysaccharides, whereas higher exopolysaccharide was noticed in KRBKKM2 in a minimal glucose medium. Both isolates were positive for siderophore production and zinc solubilization. A potassium solubilization index of 2.40 ± 1.29 and 2.10 ± 2.12 was recorded for KRBKKM1 and KRBKKM2, respectively.

### Influence of NPK bio-fertilizers on reproductive characteristics of Banana cv. Rasthali (AAB)

The application of different levels of chemical fertilizers and various formulations of bio-fertilizers on banana growth and yield.

### Effect of treatments on the mean number of fingers per hand

**Figure 5:**
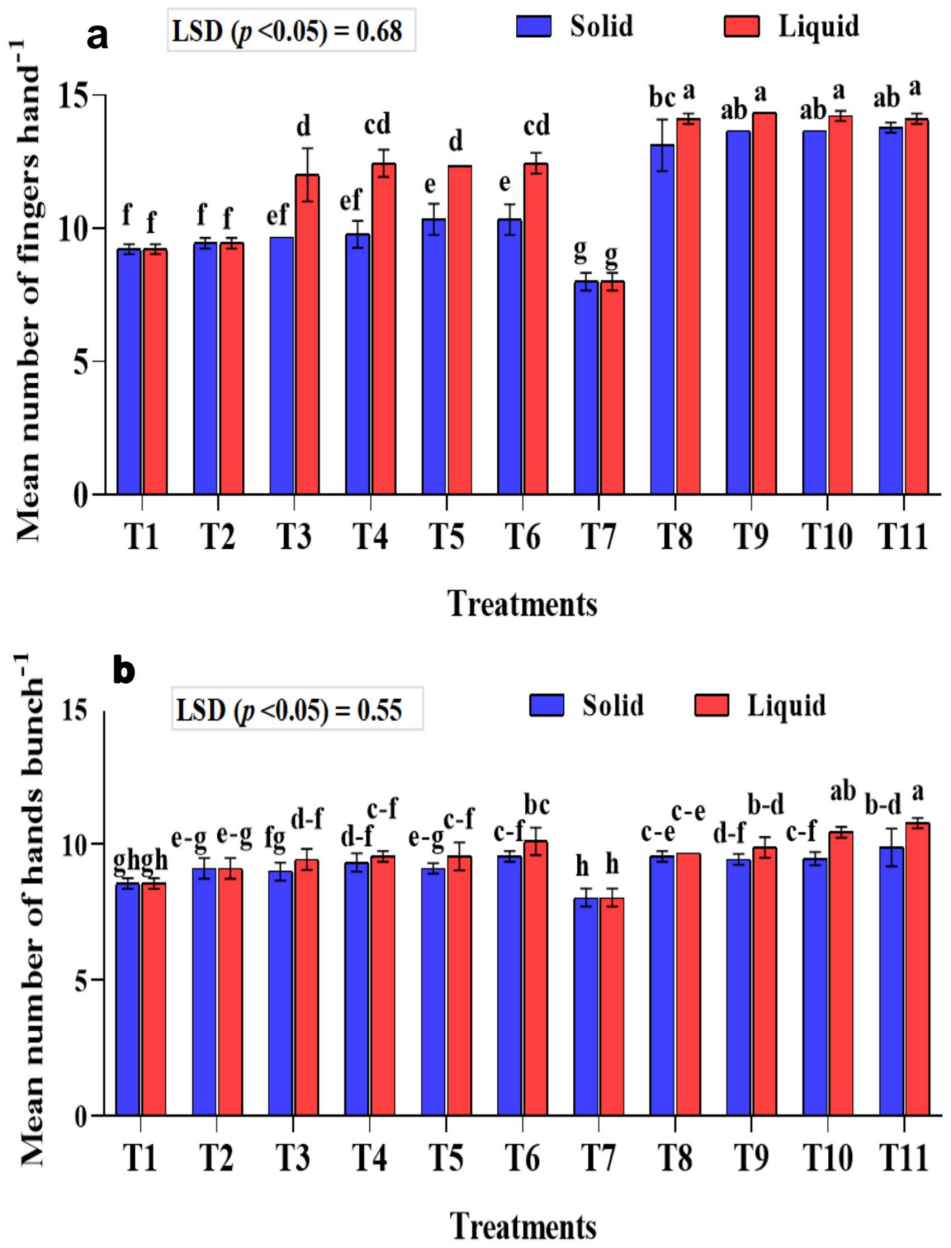
Influence of NPK bio-fertilizers on **(a)** mean number of fingers per hand and **(b)** mean number of hands per bunch of bananas. Data shown as (mean ± standard deviation, *n* = 3) followed by the different letters indicating the significant difference by the least significant difference (LSD) of the DMRT (*p* < 0.05).

The mean number of fingers per hand was significantly (*p* < 0.001) higher in the treatments that received liquid formulations than the solid-based ones (Figure 5a). Among the treatments that received liquid formulation, significantly higher mean fingers per hand of 14.11, 14.22, 14.33, and 14.11 were recorded in the T11, T10, T9, and T8 treatments, respectively. In solid-based treatments, significantly higher fingers per hand were observed in treatment plants T8-KRBKKM1; T9-KRBKKM2 with 5 g plant^−1^; T10-combination of KRBKKM 1 & 2 each with 2.5 g plant^−1^ and T11-a combination of KRBKKM 1 & 2 each with 5 g plant^−1^ along with 75% NPK and 5 g each of *A. b* Sp7 and *B. m* Pb1 than all the checks used in the study. The inoculation of KSB isolates with 75% NPK resulted in significantly higher fingers per hand than the 100% NPK used in the treatments. The interaction between formulations and treatments on the fingers was significant (*p* < 0.001).

### Effect of treatments on the mean number of hands per bunch

The mean number of hands per bunch was significantly (*p* < 0.001) higher in the liquid formulations than in solid-based ones (Figure 5b). The mean number of hands per bunch was significantly (*p* < 0.001) higher in T10 (combination of KRBKKM1 & 2 each with 1 mL plant^−1^) and T11 (combination of KRBKKM 1 & 2 each with 2 mL plant^−1^ along with 75% NPK and 5 g each of *A. b* Sp7 and *B. m* Pb1) with recorded value of 10.78 and 10.44, respectively, than all the three checks used in the study. The KSB inoculation with 75% NPK had significantly more hands per bunch than the KSB inoculation with 100% NPK. The interaction between formulations and treatments on the number of hands per bunch was non-significant.

### Effect of treatments on banana pulp weight and skin weight

**Figure 6:**
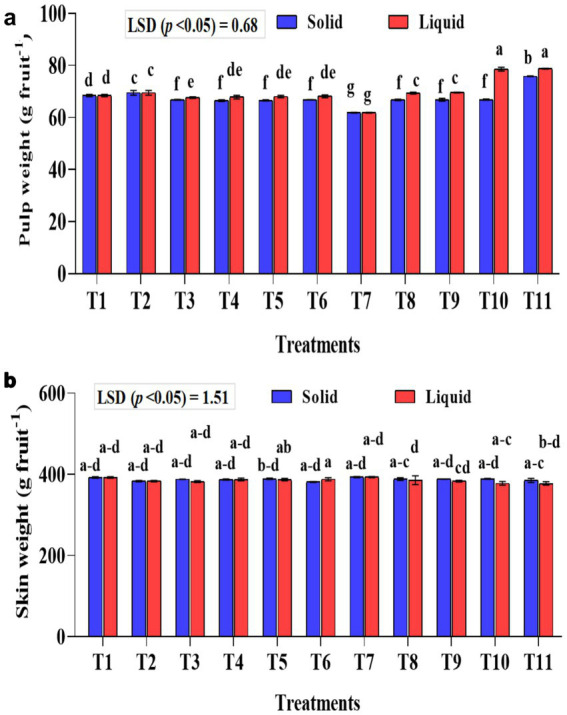
Influence of NPK bio-fertilizers on **(a)** pulp weight and **(b)** skin weight of banana fruit. Data shown as (mean ± standard deviation, *n* = 3) followed by the different letters indicating the significant difference by the least significant difference (LSD) of the DMRT (*p* < 0.05).

Significantly (*p* < 0.001) higher pulp weight was recorded with the liquid formulation than with the solid formulation (Figure 6a). In the liquid formulation, the KSB consortia with 75% NPK (T10 and T11) had significantly higher pulp weight than their corresponding 100% NPK (T5 and T6). A higher pulp weight of 78.82 and 78.61 g fruit^−1^ was recorded in T10 and T11, respectively. Regarding solid formulation, the experimental plants that received a combination of KRBKKM1 and KRBKKM2, each at 5 g plant^−1^, along with 75% NPK, and 5 g each of *A. b* Sp7 and *B. m* Pb1, recorded a significantly higher pulp weight of 75.90 g fruit^−1^. The interaction between formulations and treatments on pulp weight was significant (*p* < 0.001). The skin weight was non-significant between the formulations used and across the treatments (Figure 6b).

### Effect of treatments on the mean fruit weight and bunch weight of banana

**Figure 7:**
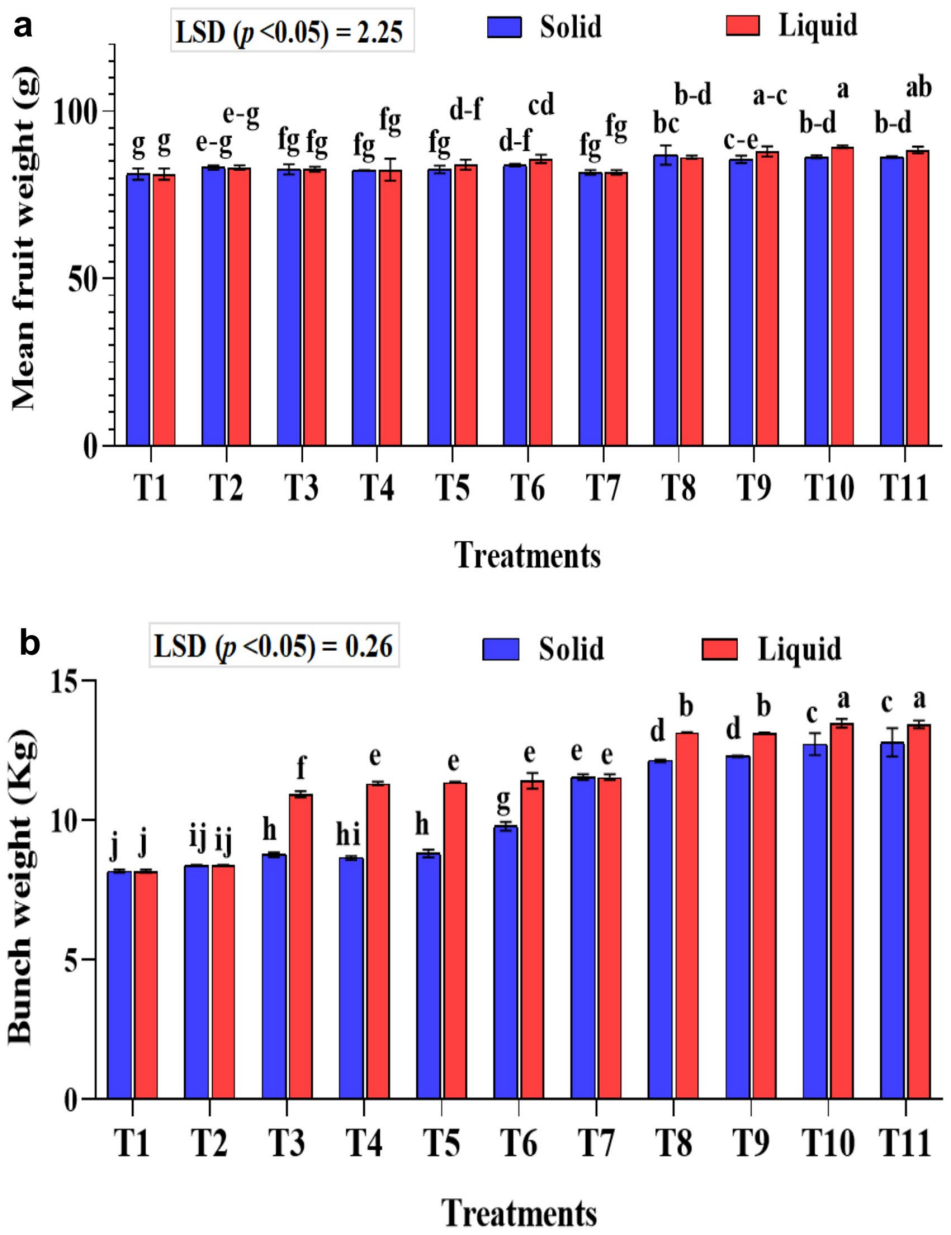
Influence of NPK bio-fertilizers on **(a)** mean fruit weight of banana and **(b)** mean bunch weight of banana. Data shown as (mean ± standard deviation, *n* = 3) followed by the different letters indicating the significant difference by the least significant difference (LSD) of the Duncan multiple range test (*p* < 0.05).

The mean banana fruit weight was significantly (*p* < 0.01) higher in the liquid formulations than the solid formulation (Figure 7a). The mean fruit weight was significantly (*p* < 0.001) higher in KSB isolates inoculated treatments either with *Agrobacterium pusense* or *Bacillus paralicheniformis* or a combination of these two along with *A. b* Sp7 and *B. m* Pb1 (T8, T9, T10, and T11) with 75% NPK used in the study in both solid and liquid formulations. Moreover, the fruit weight was significantly higher than all the checks used in the study. The mean fruit weight was significantly (*p* < 0.001) higher in 75% NPK with KSB than in 100% NPK with KSB. The interaction between formulations and treatments on fruit weight was non-significant.

The liquid-based formulation’s mean bunch weight was significantly higher (*p* < 0.001). Also, the mean bunch weight was significantly higher in the T10 and T11 treatments in both solid and liquid formulations. A significantly higher bunch weight of 13.47 and 13.43 kg was recorded in treatment plants that received 75% NPK with 2 mL each of *A. b* Sp7, *B. m* Pb1, 2 mL KRBs (T10), and 4 mL KRBs (T11). Among the solid formulations, 75% NPK with 5 g each of *A. b* Sp7, *B. m* Pb1, and either 5 g (T10) or 10 g (T11) of KRB gave a significantly higher yield than all the check treatments used in the study (Figure 7b). The inorganic NPK inputs were saved by 25% by applying these microbial consortia of NPK bio-fertilizers. The interaction between formulations and treatments on bunch weight was significantly higher (*p* < 0.001).

### Effect of treatments on the yield of banana and the total crop period

**Figure 8:**
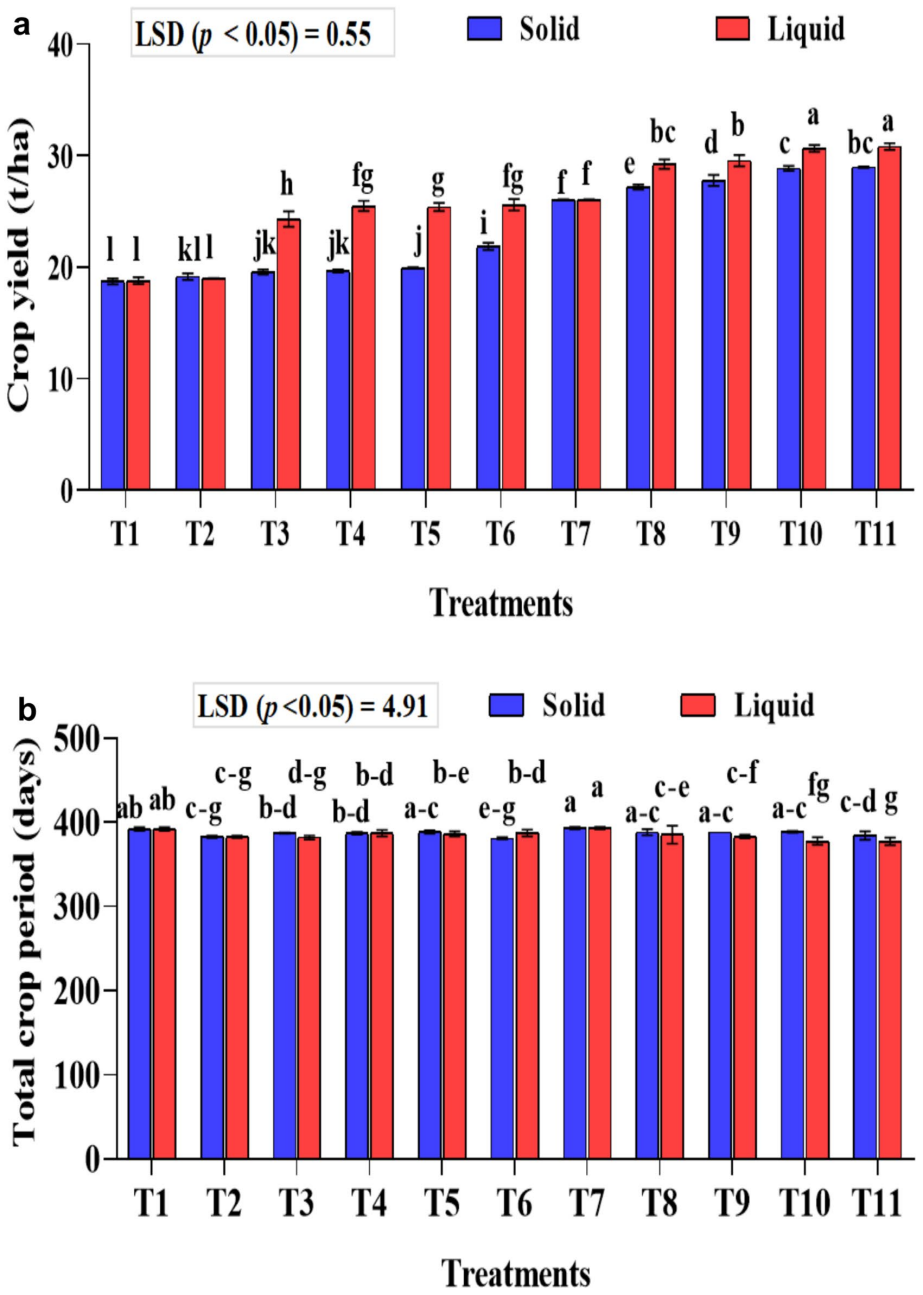
Influence of NPK bio-fertilizers on **(a)** banana crop yield and **(b)** total crop period of banana. Data shown as (mean ± standard deviation, *n* = 3) followed by the different letters indicating the significant difference by the least significant difference (LSD) of the DMRT (*p* < 0.05).

The crop yield was significantly higher (*p* < 0.001) in the liquid-based formulation than in the solid one. In solid formulation, the mean crop yield was significantly (*p* < 0.001) higher in the 75% NPK fertilizer dose along with *A. b* Sp7, phosphate solubilizing bacteria *B. m* PB1, and KSB consortium either in 5 g (T10) or 10 g (T11) quantity than all the checks used in the study and was 28.97 and 28.86 t ha^−1^, respectively. Similar treatments, such as liquid formulations, yielded 30.65 and 30.82 t ha^−1^, respectively. Regarding the solid formulation of NPK bio-fertilizers and 100% inorganic NPK, in terms of influencing crop yield (comparison between T2–T6), a yield increase of 12.44% over the control (T2) was recorded exclusively with the application of NPK bio-fertilizers. Similarly, between (T7–T12) in solid formulation with 75% NPK, an increased yield by 10.15% was noticed over the control (T7) due to NPK bio-fertilizers (Figure 8a).

About the liquid formulation of NPK bio-fertilizers and 100% inorganic NPK in influencing the crop yield (comparison between T2-T6), a 25.86% increase in yield over the control (T2) was recorded exclusively due to the application of NPK bio-fertilizers. Similarly, between (T7-T12) in liquid formulation with 75% NPK, an increased yield of 15.54% was recorded over the control (T7) due to NPK bio-fertilizers. The interaction between formulations and treatments significantly affected crop yield (*p* < 0.001).

The total crop yield was significantly shorter (*p* < 0.001) in treatments that received liquid-based formulations than in those that received solid-based formulations. The mixed KSB inoculation, at 5 g or 10 g with 75% NPK chemical doses, along with *A. b* Sp7 and *B. m* Pb1 (T10 or T11), significantly decreased the total crop period. In T10 and T11 of solid-based formulation, the crop period was decreased by 5 days and 9 days, respectively, compared to the control (T7-75% NPK + 5 g *A. b* Sp7 + 5 g *B. m* Pb1), which showed the influence of KSBs in reducing the crop period. In T10 and T11 of liquid-based formulation, the crop period was decreased by 16 days compared to control (T7-75% NPK + 2 mL *A. b* Sp7 + 2 mL *B. m* Pb1) that showed the influence of KSBs in reducing the crop period Under both formulations, the total crop period was significantly highest in the untreated control (T1) and the 75% NPK doses with *A. b* Sp7 and *B. m* Pb1 (T2 and T7) The NPK biofertilizer consortium also significantly reduced the total crop period by 100 and 75% in NPK chemical doses. The interaction between formulations and treatments during the total crop period was significantly higher (*p* < 0.01) (Figure 8b).

### Effect of treatments on potassium content in the soil and banana pulp

**Figure 9:**
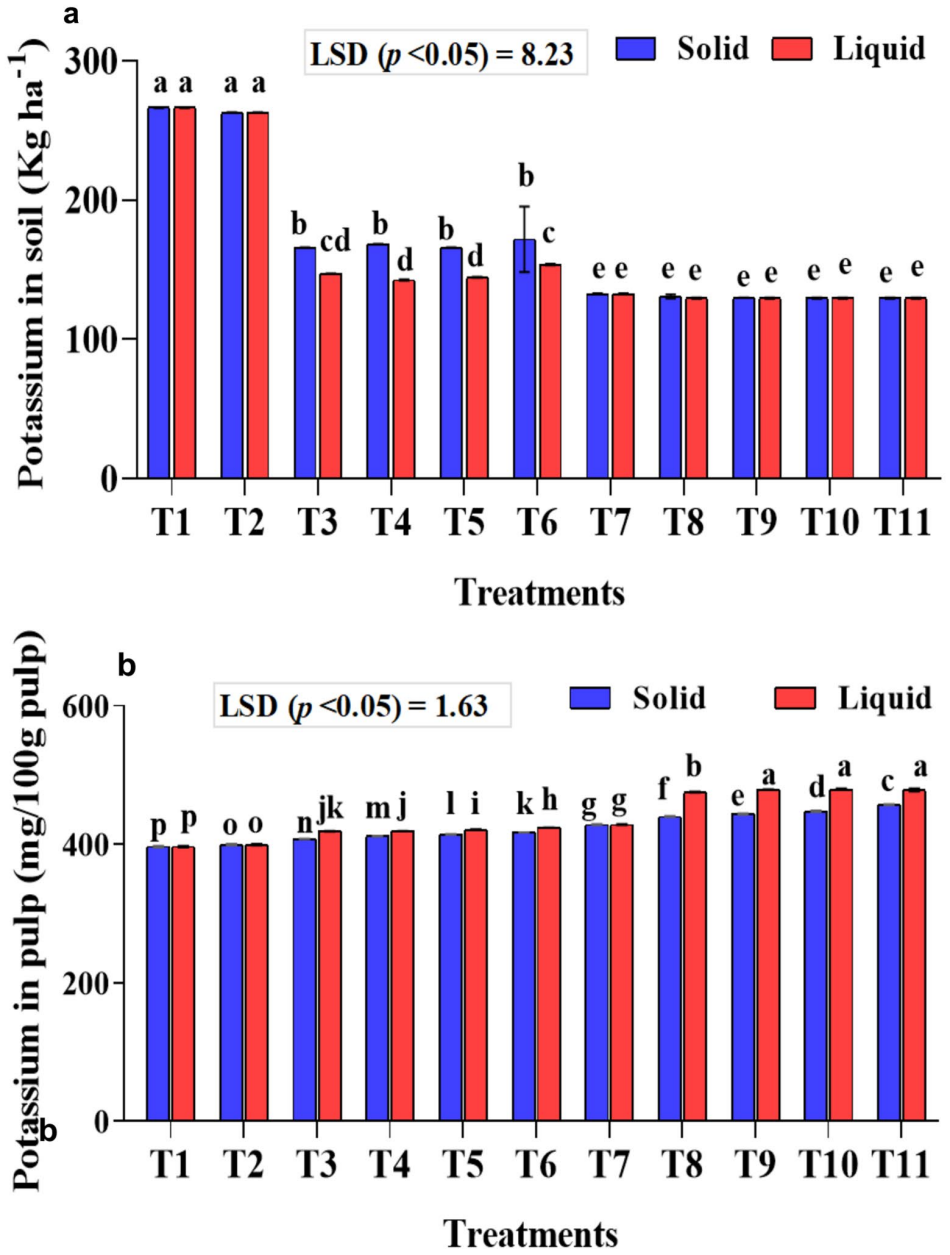
Influence of NPK bio-fertilizers on **(a)** amount of potassium in soil samples and **(b)** potassium content in banana pulp. Data shown as (mean ± standard deviation, *n* = 3) followed by the different letters indicating the significant difference by the least significant difference (LSD) of the DMRT (*p* < 0.05).

The potassium content in the soil and pulp was estimated, as the potassium requirement of bananas is higher than that of nitrogen and phosphorus. The potassium content in the soil was significantly (*p* < 0.001) lower in the soil samples that received KSB isolates along with 75% NPK. A considerably higher mean potassium content in the soil was observed with 100% NPK without biofertilizer treatment (T1-266.50 kg ha^−1^ soil). Significantly (*p* < 0.001), potassium levels in the soil were lower in samples that received NPK biofertilizers as liquid formulations compared to those that received solid-based formulations (Figure 9a). The interaction between formulations and treatments on potassium in soil was found to be significant (*p* < 0.001).

A significantly higher potassium content was quantified in the banana pulp of samples that received liquid formulations (*p* < 0.001). Similarly, significantly (*p* < 0.001) higher mean potassium in banana pulp was quantified with 75% NPK + 2 mL *A. b* Sp7 + 2 mL *B. m* Pb1 + 2 mL KRB (1 mL each of KRB KKM 1& 2-T10) and was on par with T9 and T11 treatments. The inoculation of KSB isolates, either singly or in a mixture, significantly (*p* < 0.001) improved the potassium content in banana, both in solid and liquid-based formulations (Figure 9b). The interaction between formulations and treatments on potassium in banana was also significant (*p* < 0.001).

### Principal component and Pearson correlation analysis

**Figure 10:**
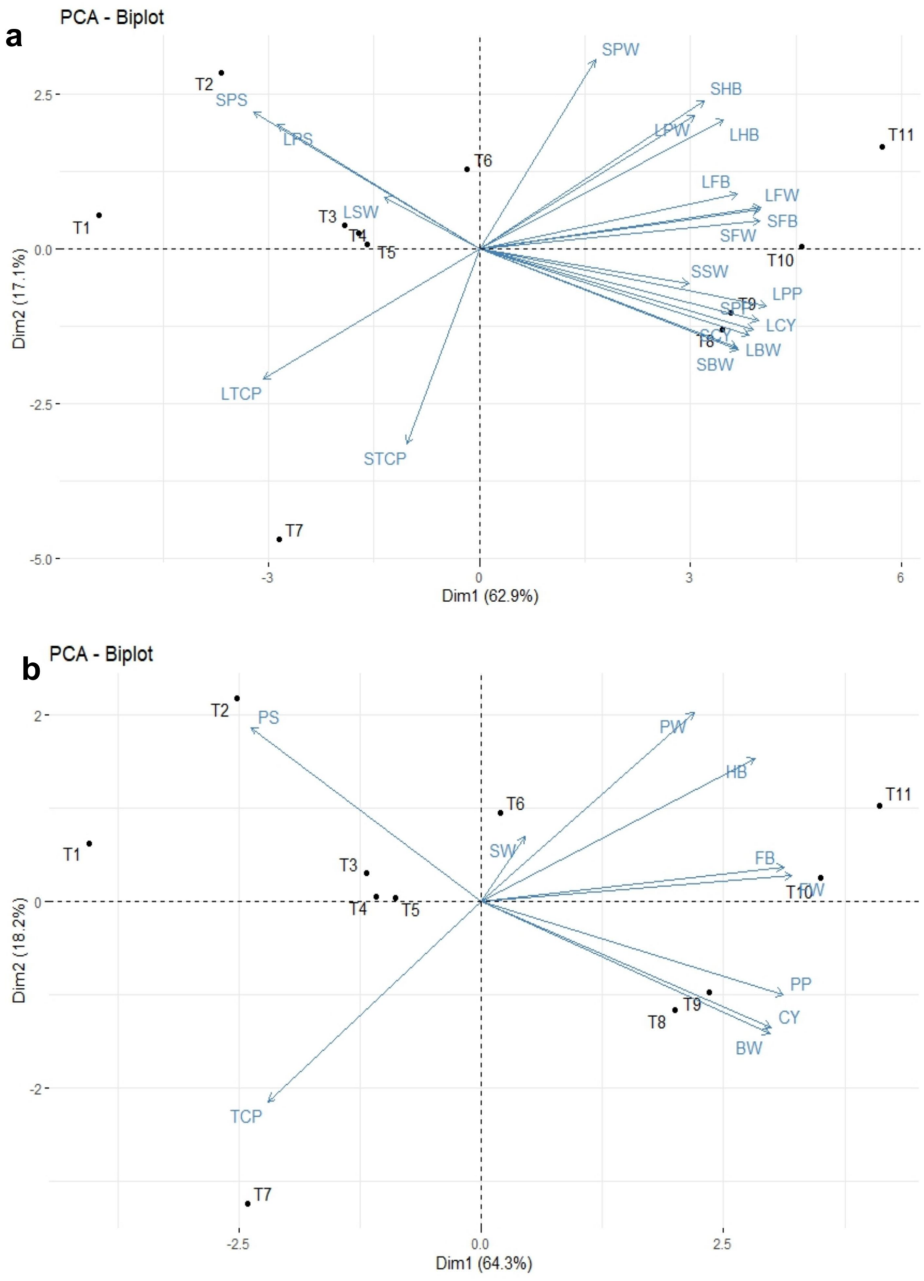
The principal component analysis of NPK bio-fertilizers that influenced various yield parameters of banana. **(a)** PCA biplot of solid and liquid microbial formulations. **(b)** Combined PCA biplot of solid and liquid microbial formulations. BW, bunch weight; CY, crop yield; SW, skin weight; HB, number of hands per bunch; FW, fingers weight; TCP, total crop period; PP, potassium in pulp; PS, potassium in soil; PW, pulp weight; CY, crop yield. Prefix SL-solid and L-liquid.

**Figure 11:**
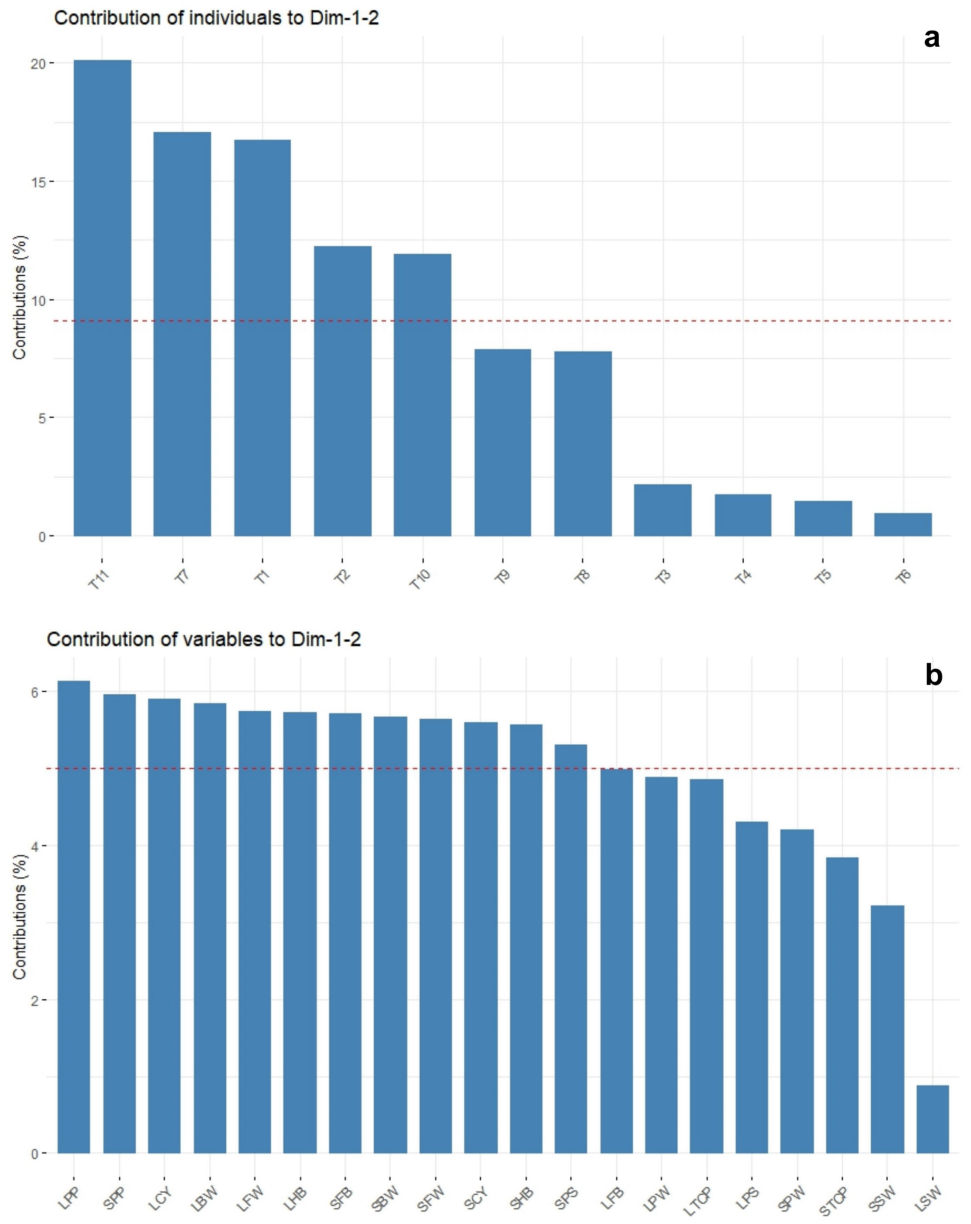
The principal component analysis depicting the influence of NPK bio-fertilizers on various yield parameters of banana. **(a)** contribution of treatments. **(b)** Contribution of yield-related parameters. BW, bunch weight; CY, crop yield; SW, skin weight; HB, number of hands per bunch; MFW, mean number of fingers per hand; TCP, total crop period; PP, potassium in pulp; PS, potassium in soil; PW, pulp weight; CY, crop yield. Prefix SL-solid and L-liquid.

The principal component analysis revealed that PC1 and PC2 accounted for 62.9 and 17.1% of the total variance, respectively (Table 5 and Figure 10a). This analysis showed that all the recorded yield-related parameters were correlated with T11, followed by T10, T9, and T8 treatments in decreasing order. In contrast, total crop period, potassium in the soil, and skin weight were correlated with T7 and T1, respectively. Among the treatments, T11 treatments showed significantly higher variance than the other treatments in both solid and liquid-based formulations. Considering both PCs, T11 followed by T7, T1, T2, and T10 treatments showed maximum variance (Figure 10b). Among the parameters recorded, the maximum variance was contributed by potassium in pulp, crop yield, bunch weight, fruit weight, and hands per bunch in both the PCs (Figure 11).

**Table 5:** Contribution of 10 different principal components (PCs) to the total variance due to the influence of NPK biofertilizers on the yield of banana and yield-related parameters.

Component	Eigenvalue	Variance percentage	Cumulative variance
Dim.1	12.58	62.91	62.91
Dim.2	3.42	17.10	80.01
Dim.3	1.91	9.59	89.60
Dim.4	0.76	3.82	93.43
Dim.5	0.50	2.53	95.97
Dim.6	0.41	2.06	98.03
Dim.7	0.29	1.47	99.50
Dim.8	0.06	0.30	99.80
Dim.9	0.02	0.12	99.93
Dim.10	0.01	0.06	100.00

Similarly, the mean PCA biplot analysis for solids and liquid-based formulations revealed 10 PCs contributing to a total variance (Table 5). Among these, PC1 and PC2 contributed 64.3 and 18.2% of the variance, respectively (Figure 10a). In PC1, T11, followed by T10 treatment, correlated with crop yield, finger weight, fingers per bunch, bunch weight, and potassium in pulp, accounting for 64.3% of the variance (Figure 10b). Similarly, among treatments, the maximum variance in both PCs was observed for T11, followed by T1, T7, T10, and T2 in decreasing order of magnitude (Figure 11a). Likewise, among recorded parameters, bunch weight, crop yield, potassium in pulp, finger weight, hands per bunch, fingers per bunch, and total crop period were in decreasing order in both PCs (Figure 11b). All the yield-contributing parameters were negatively correlated with potassium in soil and the total crop period in PC2.

**Figure 12:**
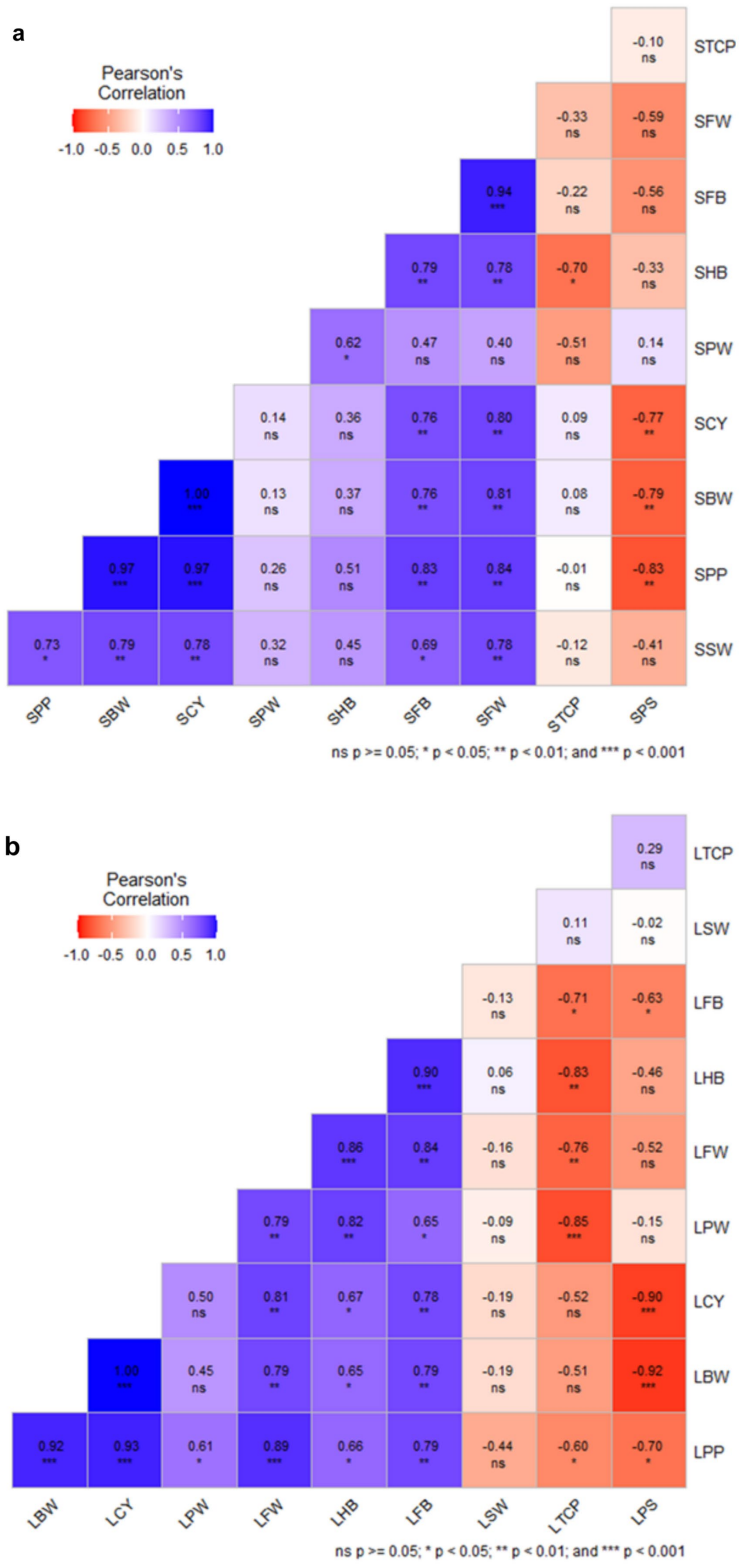
The Pearson correlation analysis of NPK solid and liquid bio-fertilizers that influenced various yield parameters of banana. BW, Bunch Weight; CY, Crop Yield; PW, Pulp Weight; FW, Fruit weight; HB, Number of Hands per Bunch; FB, Number of fingers per bunch; SW, Skin Weight; TCP, Total Crop Period; PS, Potassium in Soil; PP, Potassium in Pulp, and CY, Crop Yield. Prefix S-Solid and L-liquid. Blue shade range indicate positive correlation and that of red is negative correlation.

Pearson correlation analysis for solid-based formulation showed that crop yield is positively correlated with bunch weight (*r* = 1.00***), mean finger weight (*r* = 0.80***), and number of fingers per bunch (*r* = 0.76***). Similarly, potassium in the pulp showed a positive correlation with bunch weight, the number of fingers per bunch, mean finger weight, and crop yield. However, crop yield was negatively correlated (*r* = − 0.83***) with potassium content in the soil (Figure 12a). Similarly, correlation analysis for the liquid-based formulation showed that crop yield was positively correlated with mean finger weight (*r* = 1.00***), FB (*r* = 0.81**), the mean number of hands per bunch (*r* = 0.67*), and bunch weight. Potassium content in pulp was negatively correlated with soil potassium content (*r* = − 0.90***) (Figure 12b). Combined Pearson correlation analysis revealed that crop yield is positively correlated with bunch weight (*r* = 1.00***), mean finger weight (*r* = 0.84***), the number of fingers per bunch (*r* = 0.76***), and potassium in pulp (*r* = 0.98***). Further, crop yield was negatively correlated with potassium in soil (*r* = −0.77**), indicating that potassium was a critical element for banana yield. Therefore, the inoculation of KSB enhanced the potassium uptake and, hence, the crop yield.

### Influence of treatments and formulations on yield-related parameters

The present investigation showed that, irrespective of solid or liquid-based formulations, treatment T10 and T11 showed significantly higher values for yield-related parameters like number of hands per bunch, bunch weight, fruit weight, and crop yield (Supplementary Table S2). Two-way ANOVA between treatment and formulation showed a significant difference, except for the number of hands per bunch and fruit weight (Table 6).

**Table 6:** Two-way ANOVA of the influence of different treatments and formulations on yield-related parameters in banana.

Parameters	Treatment	Formulations	Treatment × formulation
Total crop period (days)	3.47***	1.48***	**
Number of hands per bunch	0.39***	0.16***	ns
Number of fingers per bunch	0.48***	0.20***	***
Fruit weight (g)	1.59***	0.68**	ns
Bunch weight (Kg)	0.18***	0.079***	***
Crop yield (t ha^−1^)	0.39***	0.16***	***
K in soil (kg ha^−1^)	5.82***	2.48***	***
K in banana (mg 100 g^−1^ pulp)	1.15***	0.49***	***
Pulp weight (g fruit^−1^)	0.48***	0.20***	***
Skin weight (g fruit^−1^)	1.07 ns	0.45 ns	*

*The mean difference is statistically significant at *p* < 0.05. **The mean difference is statistically significant at *p* < 0.01. ***The mean difference is statistically significant at *p* < 0.001.

## Discussion

Plant growth-promoting rhizobacteria (PGPR) enhance plant growth and development, increasing crop yields (Basu et al., 2021; Hamid et al., 2021; Sridhar et al., 2025; Ferioun et al., 2025). PGPRs exert plant growth activities that include the production of phytohormones (Khan et al., 2020), solubilization of macro and micronutrients (Sethi et al., 2025), secretion of exopolysaccharides (Ilyas et al., 2020; Budamagunta et al., 2022), and production of enzymes (Bright et al., 2025; Eswaran et al., 2024). imparting biotic and abiotic stress tolerance through ACC-deaminase activity (Sagar et al., 2020), secretion of antibiotics (Reshma et al., 2018), chitinase activity (Panicker and Sayyed, 2022), production of siderophore (Sridhar et al., 2025), and HCN production (Olaniyan et al., 2022). The present study also found that the two KSBs *Agrobacterium pusense* KRBKKM1 and *Bacillus paralicheniformis* KRBKKM2 were multi-potent. They possessed the ability to solubilize potassium and zinc, produced IAA, released siderophore, and exopolysaccharides. The quantity of potassium the microbes release would vary with the type of minerals and microorganisms at the genus, species, and strain levels. In the present study, the banana rhizosphere-borne potassium-solubilizing bacteria, *Agrobacterium pusense* KRBKKM1 and *Bacillus paralicheniformis* KRBKKM2, varied in their potassium solubilization efficiency. *Bacillus paralicheniformis* KRBKKM2 released higher potassium from mica than *Agrobacterium pusense* KRBKKM1. Both isolates were from the rhizosphere of bananas, but from different sites, and were found to differ in their efficiency. Saha et al. (2016) isolated potassium-solubilizing *Bacillus pumilus* BHU11 from the banana rhizosphere and *Bacillus licheniformis* BHU18 from the wheat rhizosphere. *Bacillus cereus* K5B, isolated from mica-rich rhizosphere soil of paddy, showed higher potassium solubilization compared to *Bacillus cereus* K5B and K6 strains from the same soil (Ghosh et al., 2023). Meena et al. (2015) reported *Agrobacterium tumefaciens* and *Rhizobium pusense* as common solubilizers of potassium from mica among other bacteria, based on 16S rDNA analysis. This study also reported *Agrobacterium pusense* KRBKKM1 (formerly *Rhizobium pusense*) as one of the promising potassium solubilizers from the banana rhizosphere.

Sarikhani et al. (2018) reported higher potassium release from biotite by *Pseudomonas* sp. strain S10-3 than from muscovite, and this study measured potassium solubilization by *Agrobacterium pusense* KRBKKM1 and *Bacillus paralicheniformis* KRBKKM2 from mica.

Regarding the mechanism of potassium solubilization, Abdoulaye et al. (2023) concluded that the release of organic acids mediated potassium solubilization by bacteria and fungi. Meena et al. (2015) reported solubilization of potassium from waste mica by the KSB *Rhizobium pusense* strain OPVS06, isolated from the sugarcane rhizosphere, through the production of organic acids. Sen et al. (2016) demonstrated that potassium-solubilizing bacteria isolated from the rhizosphere of multiple crops predominantly belonged to the *Bacillus* and *Pseudomonas* genera. The solubilization of potassium was facilitated through the production of organic acids. Tian et al. (2024) reported that the potassium solubilizer *Pantoea vagans* ZHS-1 utilized the TCA cycle, along with other pathways involving glyoxylic acid, pantothenic acid, and dicarboxylic acid to produce organic acids for the solubilization of potassium. Releasing the organic acids into the environment would lower the pH, releasing potassium ions from the minerals through protonation (Kour et al., 2020).

About the type of organic acids, *Pseudomonas aeruginosa* A10 from cotton rhizosphere exhibited higher potassium solubilization through the secretion of organic acids like lactic, citric, acetic, and succinic acids (Zhao et al., 2024). Chen et al. (2020) detected the release of formic acid, acetic acid, citric acid, and gluconate by *Bacillus aryabhattai* SK1-7 during the solubilization of potassium. Raji and Thangavelu (2021) reported the release of ascorbic, malic, and oxalic acids in varying quantities by *Bacillus subtilis*, *B. cereus*, and *B. licheniformis* during potassium solubilization. Muthuraja and Muthukumar (2022) reported acidification, by both organic and inorganic acids, as a mechanism for solubilizing potassium. HPLC analysis in this study revealed the release of propionic, succinic, and isobutyric acids by *Agrobacterium pusense* KRBKKM1, while *Bacillus paralicheniformis* KRBKKM2 released acetic, malic, tartaric, citric, propionic, fumaric, and butyric acids when grown in culture medium supplemented with mica. Among the various organic acids that have been reported for solubilization of potassium, butyric and propionic acids have not been reported in any of the KSBs, and this might be the first report of the production of butyric and propionic acids by potassium-solubilizing bacteria. Kalayu, 2019 recorded the production of isobutyric acid by *Bacillus licheniformis* and *B. amyloliquefaciens* during phosphorus solubilization.

The application of microbes as a consortium has its advantages, such as enhanced survival, additive metabolic activities, and the formation of protective biofilm structures to survive under adverse conditions. In the present investigation, inoculation of the sole or mixed KSBs along with plant growth promoters *A. b* Sp7 (nitrogen-fixer) and *Bacillus megaterium* Pb1 (phosphorus solubilizer) significantly enhanced the quality and yield-related parameters of banana. Furthermore, it saved 25% of the recommended NPK dose in banana as chemical fertilizer. The consortium-based approach would alter the rhizo-microbiome with beneficial microorganisms, thus serving as plant probiotics for enhancing crop yield (Woo et al., 2018). Previous studies have shown enhanced biomass and plant NPK content in apple seedlings when inoculating N-fixing, P and K-solubilizing *Pseudomonas* spp. (Jiao et al., 2024). In *Amaranthus*, inoculation with the nitrogen-fixer *Pseudomonas gessardi*, the P solubilizer *Bacillus* sp., the K solubilizer *Bacillus* sp., and the Zn solubilizer *Erwinia rhapontici* significantly enhanced plant biomass and biochemical parameters under both natural and controlled conditions (Devi et al., 2022). Similarly, the PGPR mix liquid formulation, consisting of *Bacillus* sp., *Azotobacter* sp., and *Azospirillum* sp., showed a 50% saving in chemical fertilizers (Gopi et al., 2020). Inoculation of a consortium of *Bacillus sonorensis* and *Funneliformis mosseae* in chilli plants showed significantly higher growth, yield, and reduced the fertilizer requirements by 50% (Thilagar et al., 2016). A previous study showed that inoculating banana plants with potassium-solubilizing bacteria (*Pseudomonas* sp.) and fungi (*Aspergillus* sp.) resulted in a 25% reduction in potassic fertilizer inputs, as well as enhanced yield parameters (Gore and Navale, 2017). Mia et al. (2010) recorded a saving of 67% of nitrogenous fertilizers in banana upon inoculating *A. brasilense* and *B. sphaericus*. These investigations demonstrated that consortium-based formulations promoted plant growth and enhanced yield by reducing the need for chemical fertilizers.

Microbial formulations determine the successful colonization of the microbes in the rhizosphere region. The present study demonstrated that liquid formulations significantly enhanced yield-related traits compared to solid-based formulations. Liquid formulations have several advantages over solid-based formulations, including enhanced shelf life, maintaining a higher population, ease of handling, thermotolerance, the ability to avoid sticky materials, the addition of microbial growth-enhancing ingredients, and ease of application (Mahanty et al., 2017). Gopi et al. (2020) showed that inoculation of liquid-based formulation of *Azotobacter chroococcum*, *Bacillus megaterium*, and *B. sporothermodurans* into the soil had greater survivability than the solid talc-based formulation at the time of harvesting the *Amaranthus* plant. A higher bacterial population of KSBs, nitrogen-fixing and phosphorus-solubilizing bacteria in the liquid-based formulations than in solid-based formulations may help in successful colonization and would have contributed to a greater banana yield.

Beneficial microbes possessing nutrient-transforming ability enhance plant growth and yield. Since bananas require a higher dose of potassium than other nutrients and are the nutrient that contributes majorly to the yield and quality of bananas, the discussion focuses on the effect of potassium-solubilizing bacteria. Inoculating KSB *Paenibacillus mucilaginosus* JGK enhanced the growth of apple seedlings by secreting phytohormones and organic acids (Chen Y. H. et al., 2020; Chen Y. et al., 2020). The KSBs belonging to *Enterobacter* sp. and *Providencia* sp. isolated from the soil samples of different crops upon inoculation in maize plants significantly enhanced the growth of seedlings, modulated antioxidant enzymes like catalase, superoxide dismutase, peroxidase, and stress enzymes like phenylalanine ammonia-lyase and polyphenol oxidase (Saheewala et al., 2023). Another study demonstrated that KSBs isolated from banana rhizosphere produced cytokinins, gibberellins, and IAA, enhancing plant growth and reducing the need for chemical fertilizers (Biswas et al., 2018).

Nutrients influence crop growth and the crop period. Islam et al. (2020) concluded that combining nitrogenous and potash fertilizers reduced the days to bunch maturity and cop duration in banana. In this study, the banana crop period was significantly reduced due to the inoculation of KSBs, *Agrobacterium pusense* KRBKKM1. *Bacillus paralicheniformis* KRBKKM2. The influence of KSBs in decreasing the crop period was prominent, resulting in a reduction of 16 days, exclusively due to KSBs used as a liquid formulation along with 75% NPK fertilizers. No earlier work is available for the reduced crop period by microbes, and this reduced crop period in this work could be attributed to the increased availability of potassium to the banana by the KSBs. Reducing the crop period in bananas would save irrigation water and help obtain a premium market price for the produce. Sun et al. (2020) recorded enhanced potassium uptake in the plants by the potassium-solubilizing *Burkholderia* from the rhizosphere of *Mikania miocrantha*.

In the present work, the soil potassium content was taken as an indicator to assess the influence of KSBs on potassium availability to the crop. It was observed that potassium content was lower in soils inoculated with potassium-solubilizing bacteria, suggesting the increased availability for utilization by the crop, reflected in the pulp’s potassium content.

## Conclusion

The usage and inclusion of biofertilizers as one of the mandatory components in agriculture is gaining importance globally. This study concluded that the consortium of *Azospirillum brasilense* Sp7, *Bacillus megaterium* Pb1, *Agrobacterium pusense* KRBKKM1, and *Bacillus paralicheniformis* KRBKKM2 could be used for enhancing banana yield and quality, with a saving of 25% of chemical fertilizer without compromising yield. Since the microbial consortium saved significant chemical fertilizers, this approach could be integrated into precision banana farming practices for enhanced productivity and economic returns. This work also demonstrated the advantages of using biofertilizers for sustainable banana farming under tropical conditions. Furthermore, it showed that the microbial consortium, formulation, and dosage could be tested on a large scale in all banana-growing areas with a tropical climate, characterized by temperatures between 22 °C and 35 °C. This targeted work, which identified specific potassium-solubilizing bacteria for banana, formulated a microbial consortium and evaluated the dosage for higher banana growth and yield, could be extended to understand the molecular mechanisms operating between banana plants and bacterial consortia. Therefore, the multi-omics study, including transcriptomics, proteomics, and metabolomics, could be explored to underpin interaction events between plants and microbes. The sustainable contribution of this microbial consortium could be validated with omics approaches.

## Data Availability

The original contributions presented in the study are included in the article/Supplementary material, further inquiries can be directed to the corresponding author.

## Footnotes

^1^ http://www.agritech.tnau.ac.in/horticulture/horti_index.html

^2^ https://www.fao.org/3/cc6952en/cc6952en.pdf

^3^ http://www.megasoftware.net/

^4^ https://agritech.tnau.ac.in/horticulture/horti_index.html

^5^ https://agritech.tnau.ac.in/org_farm/orgfarm_biofertilizers.html
