# Supplementary material for: Multi-potent rhizobacteria enhance banana growth and reduce chemical fertilizer input
Source: Front Microbiol. 2025 Sep 18;16:1659278. doi: 10.3389/fmicb.2025.1659278 (PMC12488684; doi:10.3389/fmicb.2025.1659278)
Supplement: Supplementary file 1 [file Table_1.docx]

**Supplementary Table S1.** Morphological and biochemical characteristics studied for phenotyping of the KSB isolates

| KSB isolates | Colony characteristics | Gram reaction & cell shape | Carbohydrate utilization | | | | | | Biochemical characteristics | | EPS * |
| --- | --- | --- | --- | --- | --- | --- | --- | --- | --- | --- | --- |
|  |  |  | G | F | S | L | M | C |  |  |  |
| KRB KKM1 (*Rhizobium pusense*) | Medium-sized, flat, creamish, margin irregular, light orange pigmentation on prolonged incubation | Gram-negative rod | + | + | + | - | + | - | MR | Positive | ++ |
|  |  |  |  |  |  |  |  |  | VP | Negative |  |
|  |  |  |  |  |  |  |  |  | Urease test | Positive |  |
|  |  |  |  |  |  |  |  |  | Casein hydrolysis | Negative |  |
|  |  |  |  |  |  |  |  |  | Citrate utilization | Positive |  |
|  |  |  |  |  |  |  |  |  | Catalase | Positive |  |
| KRB KKM2 (*Bacillus paralicheniformis*) | Medium-sized, raised, white, spreading, margin irregular, dark orange pigmentation on prolonged incubation | Gram- positive rod | + | + | + | + | + | - | MR | Positive | ++ |
|  |  |  |  |  |  |  |  |  | VP | Negative |  |
|  |  |  |  |  |  |  |  |  | Urease test | Positive |  |
|  |  |  |  |  |  |  |  |  | Casein hydrolysis | Negative |  |
|  |  |  |  |  |  |  |  |  | Citrate utilization | Positive |  |
|  |  |  |  |  |  |  |  |  | Catalase | Positive |  |
| Positive control-TNAU-KRB |  |  |  |  |  |  |  |  |  |  | + |

G-Glucose; F-Fructose; S-Sucrose; L-Lactose; M-Maltose; C-Cellulose

[+ presence and – absence]

*[+Low; ++ Moderate; +++ High]

**Table S2.** Influence of different treatments on yield-related parameters of banana.

| **Treatment** | **TCP (days)** | **Hands No. Per Bunch** | **Finger No. per bunch** | **Fruit weight (g)** | **Bunch weight (Kg)** | **Crop yield (tonnes ha^-^)** | **K in soil (kg/ha)** | **K banana**  **(mg 100 g^-1^ pulp)** | **Pulp weight (g/fruit)** | **Skin weight (g/fruit)** |
| --- | --- | --- | --- | --- | --- | --- | --- | --- | --- | --- |
| T1 | 392.22a | 8.55g | 9.22d | 81.23d | 8.17h | 18.75h | 266.5a | 396.52k | 68.46d | 11.64a |
| T2 | 383cd | 9.11f | 9.44d | 83.23bc | 8.38g | 19.06h | 262.8a | 399.51j | 69.5c | 11.53a |
| T3 | 384.83bcd | 9.22ef | 10.83c | 82.69cd | 9.84f | 21.94g | 156.45c | 413.17i | 67.24e | 11.64a |
| T4 | 387.22b | 9.44cdef | 11.11bc | 82.45cd | 9.98ef | 22.57f | 155.37c | 415.43h | 67.18e | 11.75a |
| T5 | 387.71b | 9.33def | 11.33bc | 83.35bc | 10.08e | 22.66f | 155.07c | 417.78g | 67.32e | 11.96a |
| T6 | 384.38bcd | 9.83bc | 11.39b | 84.9b | 10.59d | 23.73e | 162.74b | 420.49f | 67.51e | 12.44a |
| T7 | 393.33a | 8.03h | 8.0e | 81.76cd | 11.54c | 26.03d | 132.5d | 428.4e | 61.88f | 11.70a |
| T8 | 386.89bc | 9.61bcde | 13.61a | 86.61a | 12.63b | 28.21c | 130.26d | 457.66d | 68.10d | 11.43a |
| T9 | 385.55bc | 9.66bcd | 14.0a | 86.81a | 12.70b | 28.66b | 129.78d | 461.31c | 68.21d | 11.49a |
| T10 | 383.27cd | 9.95ab | 13.94a | 87.92a | 13.09a | 29.75a | 129.66d | 463.40b | 72.75b | 12.15a |
| T11 | 381d | 10.33a | 13.94a | 87.39a | 13.10a | 29.89a | 129.59d | 467.85a | 77.36a | 11.68a |
| LSD (5 %) | 4.91 | **0.55** | **0.68** | **2.25** | **0.26** | **0.55** | **8.23** | **1.63** | **0.68** | **1.51** |

Figures are the average of triplicates. Different alphabets indicate significant differences at (P>0.01)
